# Targeting food parenting practices to prevent early child obesity risk requires a different approach in families with a lower socioeconomic position

**DOI:** 10.3389/fpubh.2022.1012509

**Published:** 2022-09-26

**Authors:** Junilla K. Larsen, Levie T. Karssen, Shelley M. C. van der Veek

**Affiliations:** ^1^Behavioural Science Institute, Radboud University, Nijmegen, Netherlands; ^2^Program Group Parenting, Child Care and Development, Institute of Education and Child Studies, Leiden University, Leiden, Netherlands

**Keywords:** childhood obesity, food parenting, prevention, tailoring, socioeconomic position

## Introduction

Childhood obesity is a serious public health epidemic that occurs more frequently among children from families with a lower socioeconomic position (SEP) (1). It has generally been acknowledged that facets of the current dietary environment contribute to increased obesity vulnerability among children (2). Parents are considered a key influence in children's home food environments, particularly during early childhood (3, 4). Specifically, the home food environment is largely shaped through parents' food parenting practices (5), which refer to food-specific, goal-oriented, discrete, and observable acts of parenting (6). As dietary habits formed during early childhood may have a lifelong influence on food preferences, understanding how to promote healthy eating habits in children by influencing at-risk parents during this stage of life is very important and cost-effective (3, 7, 8). Hence, this opinion article aims to increase insight into how we can best improve food parenting practices among parents of young children from lower SEP backgrounds. To this aim, we first summarize recent food parenting practices insights from systematic reviews containing experimental, intervention, or longitudinal studies that are able to show cause-and-effect or direction of relations. Then, we will discuss high quality studies specifically examining effects of food parenting practices among parents of young children with lower SEP and consider the broader context of the potential consequences of lower SEP, because this sets the stage for intervention efforts. Finally, we will integrate and discuss these findings and provide recommendations for future research. Of note, this perspective focuses on parenting practices regarding child dietary intake, with the acknowledgment that environmental obesity influences are naturally not limited to diet.

## Food parenting practices: Insights from intervention, experimental, and longitudinal review studies

In general, three overarching dimensions of food parenting practices have been distinguished. First, structure, consisting of practices such as food rules and limits, monitoring, routines, modeling, repeated exposure, and food availability and accessibility. Second, coercive control, with practices dominating child behavior, such as restriction, threats, and instrumental or emotional feeding. Third, autonomy support, including practices that facilitate children's independence and healthy eating through for instance encouraging the child to eat autonomously, praise and non-food rewards, nutrition education, reasoning, and negotiation (9). During the past decade, reviews of experimental and (home-based) intervention studies have improved our knowledge of the influence of food parenting practices on the development of children's healthy dietary intake, although studies most often have a bias toward parents from higher SEP backgrounds. Most evidence has been found for repeated exposure to a variety of vegetables, serving a variety of vegetables, and small (non-food) rewards (10–14). Moreover, simply providing children with healthy food (i.e., availability) has been experimentally shown to affect long-term eating behavior (15). Other promising, but less examined, strategies to stimulate healthy food intake include social modeling, guided choices, portion size, and experiential learning strategies (14–16). Of note, less is known about whether and how food parenting practices may prevent children's intake of less healthy foods, while these insights may even be considered more directly important for effective childhood obesity prevention (14).

To gain more insight into the prospective links between food parenting practices and (early) children's weight outcomes we have recently provided a systematic overview of such links (17). Coercive practices, specifically restriction, pressure, and monitoring, receiving the most attention within prospective studies were generally *not* associated with children's weight outcomes over time. Instrumental feeding, and thus rewarding with food for correct behaviors, was found to be associated with higher weight over time, but more high-quality research is needed. Similarly, most autonomy supporting and structure-related food parenting practices were also important understudied constructs (17). Of note, in contrast to the longitudinal zero findings for restriction, systematic reviews (partly) based on experimental studies suggest that restriction is associated with higher intake of restricted/unhealthy foods (15, 16, 18). Future experimental studies with longer-term follow-ups may unravel these seemingly contradicting findings, taking reversed causation effects into account.

Finally, reviews of intervention studies suggest that responsive feeding is promising in the prevention of childhood obesity (19). Responsive feeding interventions stimulate child-centered and autonomy supportive food parenting practices that encourage self-regulation in eating (and discourage coercive practices) through supporting the child to eat autonomously and in response to physiological and developmental needs (20). Systematic reviews of randomized controlled trials suggest that providing responsive feeding and/or broader responsive guidance to parents compared to usual care, can stimulate more “normal” healthy weight development during infancy and preschool age (21–23). However, it should be noted that these responsive intervention studies are population-based studies that often target broader (responsive) parenting and weight-related strategies and also have a bias toward parents with higher SEP backgrounds. This SEP bias is a common trend, with many preventive (dietary) interventions not targeting young children most at risk of childhood obesity (24, 25).

## Food parenting practices in families with lower SEP: What is known?

Although studies on food parenting practices among families with higher SEP outnumber those among families with lower SEP, some relevant studies have been conducted among families with lower SEP. Most high-quality studies among parents with lower SEP have investigated feeding styles instead of food parenting practices, so direct comparisons are difficult to make. Feeding styles are usually described along the same dimensions as general parenting styles (i.e., demandingness and responsiveness) (26) but are specifically applied to the eating context, and refer to the overall context in which parents socialize their children around eating (27). To date, an indulgent feeding style (low demandingness/high responsiveness) has consistently been linked with increases in Body Mass Index z-scores over time among preschoolers living in low-income households (28–30). Remarkably, while general authoritative parenting is considered the most “healthy”—relating to numerous positive child outcomes—the authoritative *feeding* style was also related to higher child z-BMI in two out of three previously reported studies among low-income families (29, 30). Future research is needed to replicate this finding and understand what mechanisms may underlie this association. One eminent mechanism may relate to (unhealthy) food availability in the household, explaining why allowing children autonomy in what and how much they eat does not lead to healthy weight outcomes. The importance of food availability and accessibility for lower SEP families is underscored in a recent study showing that these food parenting practices were the most important ones mediating the association between parental education (i.e., important indicator of SEP) and children's dietary intake (31). Moreover, review studies suggest that healthy food modeling is also less common among families with lower SEP (32, 33). Besides, we suggest that many promising food parenting practices previously mentioned do not work equally effective for parents from lower SEP backgrounds without taking the broader perspective and SEP barriers into account, as further discussed below.

## Barriers impeding food parenting practices among parents with lower SEP

Can interventions that work in families from higher SEP automatically be assumed to work equally well in families with lower SEP backgrounds? Are determinants of food parenting practices the same in these differing groups? Are the same problems in food parenting practices present, or should we target different behavior? Families from lower SEP obviously entail a large range of diverse families, the defining features being a lower educational/occupational level of the parent(s), and/or less available income for the family, which may cause financial problems. It is well known that lower SEP is a risk factor for parental stress and lower mental wellbeing (34, 35), which each pose a risk for using more negative general parenting strategies (36, 37). Thus, families from lower SEP backgrounds may be confronted with a combination of risk factors which are likely to exacerbate each other. In the case of promoting a healthy diet, financial problems are an important barrier because unfortunately, foods of lower nutritional value still cost less per calorie and are thus more often selected by parents with lower SEP backgrounds (38). Besides food cost, lack of (nutrition) knowledge and time are often reported barriers toward healthy eating and weight status (39) that are more frequently reported among parents from lower SEP (31, 40). In addition, although parents from lower SEP, like parents from higher SEP, have more positive attitudes toward healthy food choices (41), healthfulness misperceptions are more common among “low-income” parents and appear to contribute to frequent provision of unhealthy dietary products to children (42–44). Moreover, families from lower SEP more often live in unhealthy neighborhoods with fast-food stores and less opportunities to buy healthy groceries, impacting food parenting practices, children's dietary intake, and weight development (45, 46). Taken together, it is highly likely that food parenting interventions for families with a lower SEP will require a different approach.

## Discussion and directions for future research

We therefore propose to simultaneously target three key aspects to improve food parenting practices among parents from lower SEP backgrounds, thereby “bridging” multiple socio-ecological layers at the interrelated individual, (food) environmental, and social/interpersonal level (47–50).

### Recommendation 1: Tailor to individual-level needs

A first action we propose is that, at the individual level, food parenting interventions should be tailored to the specific (mental health) needs, knowledge, and motivations of parents from lower SEP previously mentioned. Cultural diversity is also an important topic to consider, with interventions needing culturally sensitive tailoring, both regarding delivery and content (51). We even propose that tailoring the preventive approach to the needs and wishes of parents through participatory design principles is more relevant than including all evidence-based advices in terms of healthy parenting changes, as motivation is a core component that need to be fulfilled in order for a behavior change intervention to be effective (52, 53). Specifically, tailored at mental health needs, mindfulness (parenting) interventions may have great promise among some underserved (e.g., lower SEP) populations (54), as they address automatic processes underlying health (and parenting) behaviors that may particularly be important for these groups that often experience more problems with translating intentions into behaviors (55, 56).

### Recommendation 2: Make healthy food easily available

A second action we propose is to improve broader environmental-level food availability and accessibility, given the previously mentioned barriers impeding healthy child consumption. Of note, strategies focusing on tax and subsidy policies particularly benefit lower SEP groups (57). Moreover, incentives that promote healthier food purchases are rare, but may also prove promising (58). These policy changes influence broader environments and regulations, helping parents from lower SEP backgrounds to make healthy foods more easily available in their homes, facilitating important food parenting practices (e.g., healthy food availability/accessibility or modeling) that, as mentioned, are generally less common among families with lower SEP backgrounds (31–33).

### Recommendation 3: Target and deploy the social network

A final action we propose is that interventions should actively use the social context in which parents live. Parents from lower SEP may have developed greater attunement to other people and social information/relationships (59). As such, they might also be impacted more strongly by an integral approach targeting the social/interpersonal level. There is evidence that whole-of-community interventions are more effective for people with lower than higher SEP backgrounds (60). Parent support groups are appreciated by parents of young children and seem to contribute to enhancing parental knowledge, skills and practices regarding healthy behaviors, potentially benefitting young children's health behaviors (61). Moreover, a systematic review also supports the idea that interventions involving more active parental engagement strategies, such as social support, are more effective in the prevention of early childhood obesity (62). Hence, we propose that social network strengths should be more actively targeted and deployed in the field of food parenting practices (and broader obesity prevention efforts).

## Conclusion

This opinion article shows that more research is needed to examine how food parenting practices can best be targeted among “lower SEP” families. We propose that targeting structure-related food parenting practices (e.g., availability/accessibility) should have high priority among these groups. Only then, responsive (feeding) interventions may reach similar positive effects to those among parents with a generally higher SEP. Moreover, we propose that for intervening on food parenting practices among these groups, an active integral approach, “bridging” diverse socio-ecological layers, is highly important. One example to bridge the layers, is that individual-level techniques to change automatic processes underlying stress, health behaviors, and parenting behaviors are targeted at the social/interpersonal level (actions performed together with a friend or partner). Another example is to combine environmental availability of fruit and vegetables (e.g., through preschools and free provision to parents) with specific individual-level food parenting interventions. Such examples should preferably be combined, bridging all three layers. The purpose of this opinion article is to contribute to a foundation for stimulating innovative and promising lines of food parenting intervention research that actively bridge the socio-ecological layers to more effectively prevent childhood obesity among high priority populations.

## Author contributions

JL conceived the idea and wrote the first draft of the manuscript. LK and SV edited the manuscript. All authors read and approved the final version of the manuscript.

## Funding

This paper was an output from the *Samen Happie!* trial funded by a grant from Fonds NutsOhra (Grant Number: 100.939).

## Conflict of interest

The authors declare that the research was conducted in the absence of any commercial or financial relationships that could be construed as a potential conflict of interest.

## Publisher's note

All claims expressed in this article are solely those of the authors and do not necessarily represent those of their affiliated organizations, or those of the publisher, the editors and the reviewers. Any product that may be evaluated in this article, or claim that may be made by its manufacturer, is not guaranteed or endorsed by the publisher.
